# Relationship between life quality and emotional status among patients living with HIV during the second wave of the COVID-19 pandemic in Russia

**DOI:** 10.1192/j.eurpsy.2023.1720

**Published:** 2023-07-19

**Authors:** V. V. Titova, V. I. Rozhdestvenskiy, I. A. Gorkovaya, D. O. Ivanov, Y. S. Aleksandrovich

**Affiliations:** 1 Department of Psychosomatics and Psychotherapy, Saint Petersburg State Pediatric Medical University, Saint Petersburg, Russian Federation

## Abstract

**Introduction:**

Different areas of life quality are associated with emotional status. In pandemic conditions, the index of life quality may contribute to emotional stability. However, HIV-infected patients are at risk for affective disorders and are often characterized by a low life rate.

**Objectives:**

The study aimed to examine the relationship between life quality and emotional status among HIV-infected patients during the second wave of the pandemic.

**Methods:**

Data were collected between February and July 2021 using a Google form we developed. Fifty-nine HIV-positive patients participated in the study. We used the WHOQOL-BREF to examine the quality of life and the DASS-21 to determine depression, anxiety, and stress levels. Both questionnaires were adapted for use in Russia.

**Results:**

We found that 64 % of the respondents had no symptoms of depression, 61 % of the patients reported no anxiety, and 71 % had no detectable stress. We found that physical and psychological well-being was associated with depression (r_xy_ = -0.318, p < 0.05) and anxiety (r_xy_ = -0.308, p < 0.05), microsocial support was associated with depression (r_xy_ = -0.430, p < 0.01) and anxiety (r_xy_ = -0.330, p < 0.05), social well-being with depression (r_xy_ = -0.375, p < 0.01), anxiety (r_xy_ = -0.448, p < 0.01) and stress (r_xy_ = -0.362, p < 0.01).

**Conclusions:**

During the second pandemic wave, the social well-being was most strongly associated with emotional well-being among patients living with HIV. This indicates that different types of social support are essential for this group of patients. Therefore, state authorities should pay special attention to the social welfare of this group of patients.

**Disclosure of Interest:**

None Declared

